# Acute Cholecystitis Presenting With Septic Shock as the First Presentation in an Elderly Patient

**DOI:** 10.7759/cureus.20981

**Published:** 2022-01-06

**Authors:** Ahmed Auda, Rashid Al Abdullah, Mohammed O Khalid, Wedyan Y Alrasheed, Sumaiyah A Alsulaiman, Fai T Almulhem, Meriam F Almaideni, Aisha Alhikan

**Affiliations:** 1 General and Laparoscopic Surgery, Prince Saud Bin Jalawi Hospital, Al Ahsa, SAU; 2 Infectious Disease, Prince Saud Bin Jalawi Hospital, Al Ahsa, SAU; 3 General Surgery, Prince Saud Bin Jalawi Hospital, Al Ahsa, SAU; 4 Medicine, King Faisal University, Al Ahsa, SAU

**Keywords:** percutaneous cholecystostomy tube, biliary system, gallbladder, gallstone disease (gsd), gangrenous cholecystitis

## Abstract

Gangrenous cholecystitis (GC), a severe complication of acute cholecystitis, is associated with higher morbidity and mortality rates than uncomplicated cholecystitis. In this report, we present the case of an 81-year-old female with diabetes mellitus and hypertension who presented in the emergency department complaining of severe generalized abdominal pain for 10 days. The pain was associated with nausea and vomiting. She had septic shock, prompting admission, and was eventually diagnosed with perforated GC. Interventional radiology was conducted, and a cholecystostomy tube was placed under radiology guidance with continuous daily irrigation and intravenous antibiotic coverage for four weeks. Subsequently, the patient’s condition improved, and she was finally discharged.

## Introduction

The biliary system is mainly composed of the gallbladder, cystic duct, common bile duct (CBD), and sphincter of Oddi. Bile, which consists of bile salts, cholesterol, and bilirubin, is the main liquid that flows in the biliary system. When the gallbladder malfunctions, bile becomes supersaturated, turning into gallstones [1].

Gallstone disease is the most common disorder of the biliary system [2], with a prevalence of 10-15% in adults [3]. However, its prevalence varies by ethnicity. For example, Native Americans have the highest prevalence, whereas Black Africans have the lowest. There are several types of gallstones according to their components such as pure cholesterol, pure pigmented, and mixed stones [4]. The risk factors for gallstone development are either modifiable or nonmodifiable. Modifiable risk factors include metabolic syndrome, dietary factors (high-calorie, high-carbohydrate, and low-fiber diets), certain diseases (liver cirrhosis and Crohn’s disease), ileal resection, and certain medications (hormone replacement therapy and octreotide). Conversely, nonmodifiable risk factors include female gender, advancing age, race, and lithogenic genes [3].

Gallstone disease can be asymptomatic. Its symptoms range from mild to severe pain or complications [5]. The cardinal symptoms of gallstone and biliary system diseases are right-upper-quadrant or epigastric colicky pain, jaundice, and fever. In addition, gallstone disease has several presentations. Biliary colic occurs when abdominal pain becomes recurrent and lasts longer than 15 minutes. The pain can radiate to the right shoulder or the back and can be associated with nausea and vomiting [6].

Moreover, gallstone disease is associated with multiple complications, including biliary colic, acute cholecystitis, gallstone pancreatitis, obstructive jaundice, and ascending cholangitis secondary to choledocholithiasis [7]. Acute cholecystitis is the most frequent [6] and occurs when the gallstone blocks the cystic duct, causing gallbladder inflammation. Acute cholecystitis usually presents with the typical abdominal pain lasting for more than six hours and a history of biliary colic, fever, and Murphy’s sign, which occurs when the patient stops breathing due to pain with deep right-upper-quadrant palpation. Furthermore, laboratory workup may also show leukocytosis and mild bilirubin elevation. The recommended diagnostic tests include ultrasonography or hepatobiliary iminodiacetic acid scan and computed tomography (CT) when complications are suspected [7]. Although gallstones are the most common cause of acute cholecystitis, dysfunction or hypokinesis of gallbladder emptying can also cause acute cholecystitis, which is called acalculous cholecystitis. Acalculous cholecystitis is commonly associated with total parenteral nutrition, long-term fasting, and extreme weight loss. Its risk factors include intensive care unit (ICU) admission, myocardial infarction, severe burns, sepsis, stroke, and severe trauma [8].

In some patients with diabetes mellitus, acute cholecystitis can be complicated because of masked symptoms and delayed presentation, which can progress to gangrenous cholecystitis (GC) and perforation, leading to peritonitis. Consequently, morbidity and mortality increase. GC has several risk factors such as old age, diabetes mellitus, male sex, leukocytosis, and coronary heart disease [9]. Although gallbladder perforation is a rare complication of acute cholecystitis, it can be life-threatening, with a mortality rate of 12-42% [10]. This case report aims to outline the stepwise approach toward an elderly patient who presented with septic shock secondary to perforated GC as well as the subsequent definitive management to minimize morbidity and mortality in this age group.

## Case presentation

An 81-year-old Saudi female with diabetes and hypertension presented to the emergency department (ED) with complaints of severe generalized abdominal pain for 10 days. The pain intensity had increased over time in the right upper quadrant region. She described the pain as dull and vague. The abdominal pain was associated with nausea, anorexia, multiple greenish vomits, and subjective on and off fever. Five days prior, she had suffered from constipation. On presentation, she was conscious but confused and disoriented to time, place, and people. She looked ill and appeared to be in severe pain, but no icterus or cyanosis was observed. Her vital signs revealed an unstable condition, as shown in Table 1.

**Table 1:** Vital signs of the patient upon admission.

Vital sign	Patient’s measurement
Blood pressure	90/50 mmHg
Heart rate	120 beats per minute
Breathing rate	22 cycles per minute
Oxygen saturation	96%
Temperature	38°C

Cardiac examination showed tachycardia with normal first and second heart sounds and no additional sounds. On pulmonary examination, the patient had dyspnea. Moreover, air entry was bilaterally decreased but was more intense on the right side. Abdominal examination findings revealed a distended abdomen, generalized tenderness, guarding in the right upper quadrant region, no bowel sounds, and positive Murphy’s sign. In the ED, the patient received resuscitation protocol, which included O_2_ therapy, pulse oximetry, chest examination to exclude atelectasis, and good venous access insurance. A blood sample was drawn for complete blood count, biochemical profile, coagulation profile, arterial blood gases, and blood group assessment. Hour-1 Bundle of Surviving Sepsis Campaign (SSC) was initiated to measure the serum lactate levels and obtain a blood culture before the administration of antibiotics. Subsequently, we initiated piperacillin/tazobactam as broad-spectrum antibiotics. Although we rapidly administered 20 mL/kg of crystalloid bolus, the patient remained hypotensive and tachycardic. Therefore, she was admitted immediately to the ICU where she received 5 µg of noradrenaline. Upon initial laboratory evaluation, an increase in white blood cells was noted, as shown in Table 2.

**Table 2:** Laboratory findings during admission.

Test	Patient’s result	Normal range
White blood cells (×10^9^/L)	20.60	4–11
Hemoglobin (g/dL)	11.10	13–17
Platelet count (×10^9^)	437	130–400
Urea (mmol/L)	13	1.7–8.3
Creatinine	73	53–120
Aspartate aminotransferase (U/L)	15	0–40
Alanine aminotransferase (U/L)	13	30–65
Lactate dehydrogenase (U/L)	225	100–200
Alkaline phosphatase (IU/L)	101	50–135
Total bilirubin (µmol/L)	15.10	0–24
Direct bile (µmol/L)	5.76	0–5
Amylase (U/L)	45	20–115
Albumin	17.6	30–50
Fasting glucose (mmol/L)	6.70	4.1–8.3

**Figure 1:**
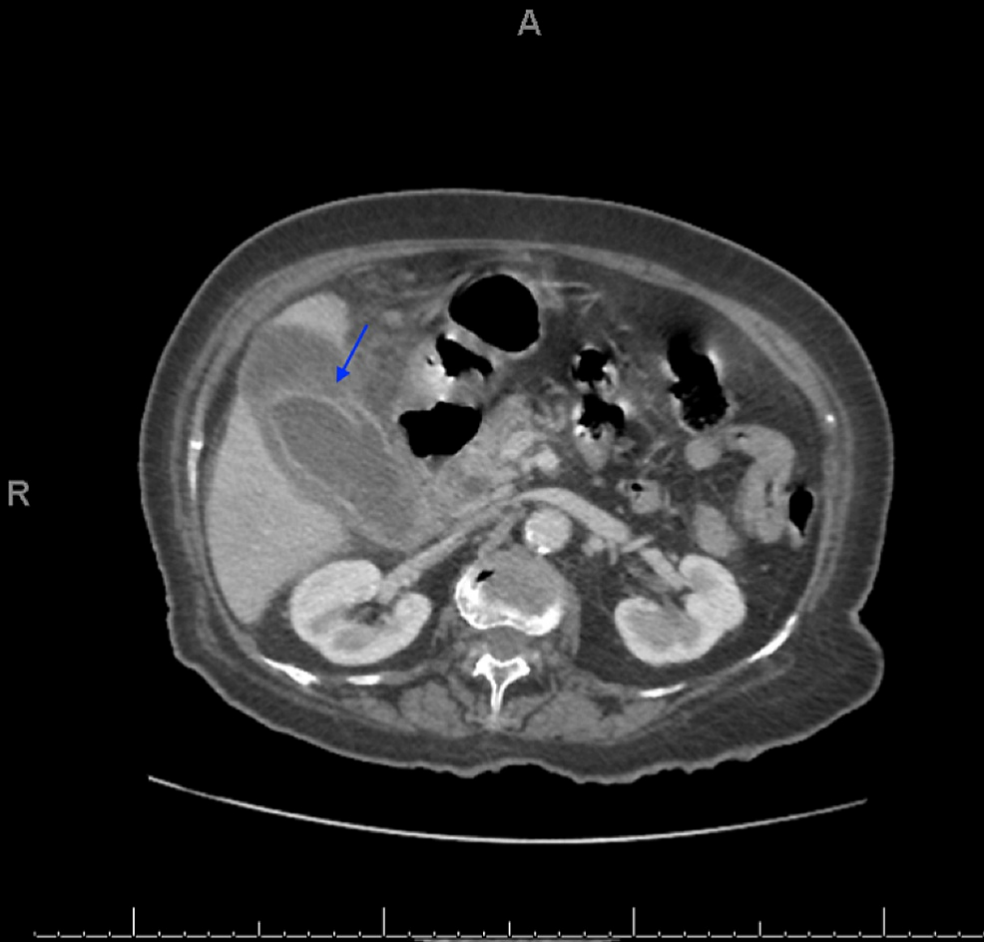
CT scan of the abdomen and pelvis with intravenous contrast in the portal venous phase. CT: computed tomography

Once the patient was stabilized, abdomen and pelvic CT with contrast was performed. The gallbladder wall was significantly thickened and surrounded by free fluid and fat stranding. No gallstones were detected. A dilated CBD (9.9 mm), wall defect, and pericholecystic collection were noted along the anterior wall of the gallbladder approximately measuring 5 × 3.8 cm (Figure 1). Blood culture and sensitivity showed growth of *Streptococcus anginosus* and *Streptococcus constellatus* (Viridans group), which are sensitive to ampicillin, augmentin (amoxicillin and clavulanate), and penicillin.

**Figure 2:**
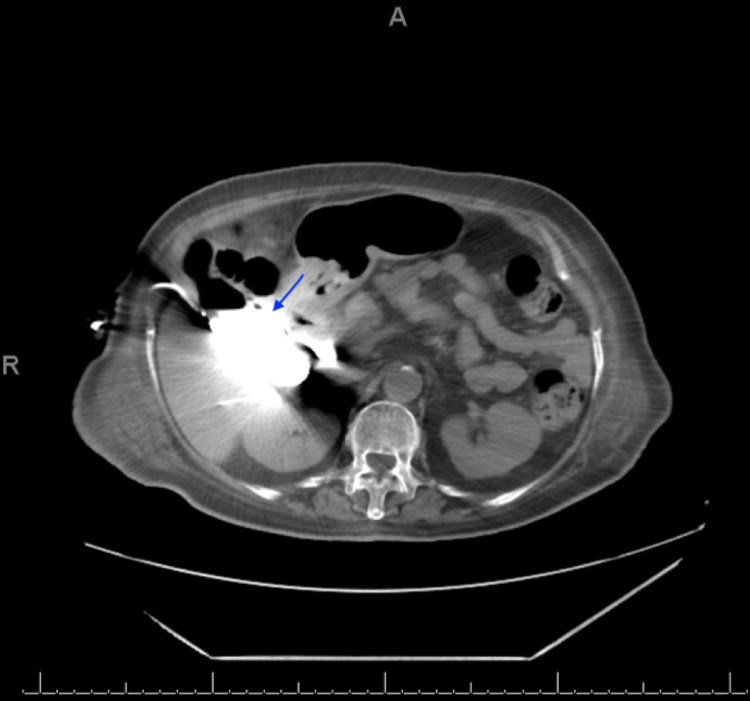
CT scan of the abdomen without intravenous contrast. The contrast was injected through the cholecystostomy tube. CT: computed tomography

Subsequently, the patient was taken immediately to the ICU to complete the SSC. A central line was placed in addition to a Foley catheter for hourly input/output recording. Low-molecular-weight heparin (40 mg) was also administered once daily for deep vein thrombosis prophylaxis. An interventional radiologist was contacted for the placement of the cholecystostomy tube. Six hours after the ICU admission, the cholecystostomy tube was placed under CT guidance in the radiology department and 200 mL of pus was aspirated. After two days, the drain output became zero, and the parameters improved. Therefore, the patient was transferred to the general ward where she was maintained on a 20-30 mL sterile saline irrigation through the tube daily. Subsequently, the irrigation was discontinued, and the antibiotic therapy was changed to imipenem/cilastatin (Tienam) according to blood culture. Over three weeks, all parameters normalized, and the cholecystostomy tube started to drain bile. After four weeks, a cholecystogram with contrast through the cholecystostomy tube was conducted, which flowed freely. Then, it opacified the gallbladder, posterior portion of the intrahepatic biliary ducts, cystic duct, and common hepatic duct, eventually reaching the duodenum. No contrast leak or intra- or extrahepatic biliary duct dilatation was observed. The gallbladder collection showed interval improvement, but multiple air pockets within the organ were still noted (Figure 2). Thereafter, the cholecystostomy tube was removed, and the patient was discharged in a stable condition.

## Discussion

Our patient was tachycardic and hypotensive for which she needed resuscitation, stabilization, empirical antibiotics, and ICU admission. Although the diagnosis of acute cholecystitis was clear from the patient’s history and examination with a strongly positive Murphy’s sign, we collected multiple blood cultures, urine culture, and throat swabs for culture and sensitivity tests to rule out other infections for treating the patient. Another important point to highlight in this case is that the late presentation (after 10 days) of abdominal pain, the patient’s old age, negligence, poorly controlled diabetes, the diabetic complication of neuropathy, and/or other comorbidity caused the patient’s perforated gallbladder and septic shock.

Given that the presentation of generalized abdominal pain in older patients is vague, the diagnosis can be difficult and includes many differential diagnoses, such as acute mesenteric ischemia, abdominal aortic aneurysm, bowel obstruction, diverticular disease, appendicitis, peptic ulcer disease, biliary disease, and pancreatitis, as well as other nonabdominal causes of abdominal pain, such as myocardial infarction, diabetic ketoacidosis, pulmonary diseases involving the lower lobes, and genitourinary issues [11]. Physicians should be aware of these broad differentials and fully assess patients to rule them out. In addition, as in this case, biliary diseases in the elderly age group usually present with complications such as gallbladder gangrene, gallbladder perforation, emphysematous cholecystitis, sepsis, choledocholithiasis, cholangitis, gallstone pancreatitis, and gallstone ileus [12].

In all previously reported cases of perforated GC, the patients were older (>65) and showed a cholecystitis complication (perforated or gangrenous gallbladder) as the first presentation; however, all of them were stable at presentation [9,13-15]. Conversely, our patient exhibited septic shock as the first presentation. This report agrees with other similar case reports that highlight poorly controlled diabetes as a cause of late presentation complicated with cholecystitis and perforated gallbladder. Poorly controlled diabetes may obscure the patient’s symptoms [14,15].

Laparoscopic cholecystectomy is the gold standard management of acute calculus cholecystitis and should always be considered as the first option, except in cases of absolute anesthetic contraindications and septic shock [16]. The alternative for surgical management is percutaneous cholecystostomy, which can be considered in the management of patients older than 65 years who are deemed unfit for surgery because of severe comorbidities. This alternative should also be considered for patients at high risk for general anesthesia and those manifesting septic shock. In performing percutaneous cholecystostomy, percutaneous transhepatic cholecystostomy is preferred [16]. In older patients with complicated acute cholecystitis, broad-spectrum antibiotic regimens are recommended and should be based on the most frequently isolated pathogens. In biliary infection, the most often isolated pathogens are Gram-negative aerobes, such as *Escherichia coli* and *Klebsiella pneumonia*, and anaerobes, especially *Bacteroides fragilis* [16]. Because our patient was older and presented with septic shock, cholecystectomy was not the ideal option. Therefore, we opted for percutaneous cholecystostomy tube placement with empirical antibiotic coverage as the definitive management.

## Conclusions

GC is a detrimental complication of acute cholecystitis with a mortality rate of 12-42%. Its risk factors include old age, male sex, poorly controlled diabetes, coronary artery disease, and leukocytosis. In cholecystitis, for patients with diabetes and/or coronary artery disease who present with signs of septic shock, ruling out GC should be considered. As in this case, the patient was elderly and presented with septic shock, the rapid resuscitation and stabilization to save her life is the major point to be highlighted. In addition, high clinical suspicion is needed to diagnose such a case of abdominal pain in the elderly and to find the most appropriate surgical or nonsurgical management approach for this age group.
